# Identifying accurate link predictors based on assortativity of complex networks

**DOI:** 10.1038/s41598-022-22843-4

**Published:** 2022-10-27

**Authors:** Ahmad F. Al Musawi, Satyaki Roy, Preetam Ghosh

**Affiliations:** 1 Department of Information Technology, University of Thi Qar, Thi Qar, Iraq; 2 Department of Genetics, University of North Carolina, Chapel Hill, NC USA; 3 Department of Computer Science, Virginia Commonwealth University, Richmond, VA USA

**Keywords:** Computer science, Computational science, Computational biology and bioinformatics

## Abstract

Link prediction algorithms in complex networks, such as social networks, biological networks, drug-drug interactions, communication networks, and so on, assign scores to predict potential links between two nodes. Link prediction (LP) enables researchers to learn unknown, new as well as future interactions among the entities being modeled in the complex networks. In addition to measures like degree distribution, clustering coefficient, centrality, etc., another metric to characterize structural properties is network assortativity which measures the tendency of nodes to connect with similar nodes. In this paper, we explore metrics that effectively predict the links based on the assortativity profiles of the complex networks. To this end, we first propose an approach that generates networks of varying assortativity levels and utilize three sets of link prediction models combining the similarity of neighborhoods and preferential attachment. We carry out experiments to study the LP accuracy (measured in terms of area under the precision-recall curve) of the link predictors individually and in combination with other baseline measures. Our analysis shows that link prediction models that explore a large neighborhood around nodes of interest, such as CH2-L2 and CH2-L3, perform consistently for assortative as well as disassortative networks. While common neighbor-based local measures are effective for assortative networks, our proposed combination of common neighbors with node degree is a good choice for the LP metric in disassortative networks. We discuss how this analysis helps achieve the best-parameterized combination of link prediction models and its significance in the context of link prediction from incomplete social and biological network data.

## Introduction

A wide range of real-world problems can be effectively solved by modeling them as complex networks which are represented as a graph having nontrivial topological characteristics compared to random networks^1^. Such complex networks play a significant role in identifying the significance of nodes and understanding the different connectivity patterns using different algorithms.

Existing algorithms on complex networks can answer different questions such as ranking the nodes based on some characteristics, predicting the structures of different topologies, and showing the flow of node/edge influence within the network among others. Link prediction (LP) models form an important class of such algorithms for complex networks. They are widely used on social networks (like Facebook, Twitter, LinkedIn, YouTube, etc.) as a way for suggesting friends, groups, videos, and any sort of possible group affiliation. In recommendation systems (such as books, movies, music, etc.), LP models were used to improve the similarity measurement of collaborative filtering methods, by exploring the association within user-item interactions to predict user interests and preferences^2^. For example, they have been used to promote products for people who share the same shopping behavior e.g., on Amazon, or promote movies on YouTube or Netflix and so on. Similarly, in biological systems, the high-throughput methods often detect an incomplete view of the network and exhibit high false-positive and false-negative rates of protein interactions^3^. LP models play a key role in predicting the possible Protein–Protein interactions in the biological system^4^. Other applications are collaborative prediction in scientific co-authorship networks^5^, predicting the spread of epidemic disease, detecting the drug-target interactions for drug discovery^6,7^ and even in bio-inspired networking^8–11^.

The prediction of the future structure, or more precisely a future edge in a complex network is considered an active and ongoing research area that affects several applications in network science. Apparently, the entry of a new node (or set of nodes) into the network or the creation of new edges (or removing edges) is applicable to many network applications. Therefore, the prediction of future links plays a critical role in understanding the future structure of the network and hence its effect on the functionality of the entire network or on a particular section of it. It is essential to reveal the mechanism by which a new (or an existing) node makes a connection with another, considering the difference in functionality and structure of the different networks. Hence, link prediction in complex networks has received wide attention using different approaches, such as graph theoretical methods, machine learning methods, probabilistic and statistical methods and so on^12,13^. Among them, the graph theoretic approach is considered a favorable way of computing the possible future links in comparison with the other methods, due to its general applicability. It uses the different graph features of the network to score possible future links between two given nodes. These methods consider the similarity between the two nodes as a way of determining the future link(s). Existing similarity-based methods can be classified into three categories: (1) local approaches, (2) global approaches and (3) quasi-local methods. We mainly focus on the local approaches due to their high performance and accuracy. In local similarity-based approaches, the nodes’ local structure (neighborhood) is the key metric used to compare different nodes. Local approaches are faster than the rest of the methods as they only require a scan of one layer of the neighborhood for each comparison. Several methods exist that measure the similarity of two nodes based on their local information.

Due to the complexity exhibited in the structures of complex networks, several algorithms measure either the overall network properties (like the degree distribution, clustering coefficient, hierarchical structures, and so on) or rank a network’s nodes by measuring the node’s influence or contribution in the network (e.g., different centrality measures such as degree, eigenvector, PageRank, betweenness^14^ and so on^15^). Another important metric is the *assortativity* of the network^16–18^—a network property that refers to the preference of the nodes to affiliate/connect with other nodes that share similar features. Such features could refer to the similarity in degree, neighborhood, the existence of shared shortest paths, and so on. Specifically, a network is said to be *assortative* if its high-degree nodes connect with other high-degree nodes and low-degree nodes connect with other low-degree nodes. For example, in social networks (like Facebook), a node (or a person) has a high chance to make a relationship with others that share the same set of nodes (friends). Conversely, a network is said to be *disassortative* if its high-degree nodes connect with low-degree nodes. For example, biological networks exhibit such connectivity patterns where nodes with low-degree tend to have a connection with high-degree nodes.

### Contributions

In this paper, we make the following contributions. First, we propose three sets of link prediction models based on (1) the similarity, (2) the dissimilarity of neighborhoods, and (3) extended preferential attachment^19^ models—all using local topologies of the two nodes. We did not consider other path dependent metrics (such as betweenness^14^, closeness^20^, average shortest path etc.). Instead, we considered the global and local influence of a node relative to the network as a measure of the node’s participation (or influence) within the network. Second, we propose a model for link prediction for a missing edge (*x*, *y*) using a parameterized combination of two different methods and compare their different versions against the assortativity value. In course of the analysis, we employ a combination of *common neighbors method* and each one of the three prediction models (similarity, dissimilarity, and extended preferential attachment). The common neighbor method is selected by default in the parameterized combination model due to its simplicity, intuitiveness, and performance. Also, it has earlier shown very competitive results in comparison with many complex approaches on real-world networks^13^. It is possible that two nodes establish a connection if they are similar in relationships (i.e., share the same neighborhood) and both nodes provide a relatively better influence within the network. In this context, *similarity* of two nodes (*x*, *y*) refers to the degree of similarity between neighbors of nodes *x*, *y*, while *influence* refers to the degree of connection (number of edges) that a node has. We make several modifications to measure a node’s influence with regard to its neighbors’ influence and the average network influence.

Third, and most importantly, we explore how the accuracy of the link prediction models varies with the assortativity of the networks. To this end, we present an approach that adapts an existing assortative network generation algorithm by Zhou et al.^21^ to create networks of varying levels of assortativity (and disassortativity). We use this approach to determine the best-parameterized combination of link prediction models. Finally, we carry out extensive experiments on real-world and synthetic networks to evaluate the proposed link prediction models against standard local similarity-based algorithms and well-studied link prediction metrics taken from the literature. We show that similarity-based models perform better in highly assortative networks where a large percentage of edges connect nodes having a similar degree to each other. On the contrary, the dissimilarity-based models perform better in highly disassortative networks where nodes tend to form connections with other nodes that are dissimilar.

## Material and methods

### Dataset

We employ network data from the following sources to validate the proposed link predictors. (Table 1 shows the essential statistics for each of the selected networks.)

1. *Karate*^22,23^ is a network of 34 members of a Karate club that reflects the members’ state of affiliation into groups due to a conflict between administrators and instructors. The dataset was collected and studied by Wayne W. Zachary from 1970 to 1972.
2. *Dolphins*^23,24^ is a network that represents the frequent associations among 62 bottlenose dolphins.
3. *Polbook* is a network of US politics books (as nodes). Edges represent the frequent co-purchasing of books on amazon.com by the same buyer. The network dataset was retrieved from http://www.orgnet.com/.
4. *USAir*^25^ is a network of airports (as nodes) and airlines (as edges) that represents the US air transportation system connecting the US around the globe.
5. *Word Adjacency*^23,26^ is a network that represents the existence of either noun-noun, adjective-noun, or adjective-adjective adjacent words in the novel of “David Copperfield”. Nouns and adjectives are represented as nodes, and their adjacency is represented as edges.
6. *Escherichia Coli GRN*^27^ is a biological network that reflects the genes and transcription factors of E. Coli and how they interact with each other to regulate the functionality of the organism. Genes and transcription factors represent the nodes and their interactions represent the edges of the network.
7. *Barabasi-Albert*^28^ is an algorithm that generates random scale-free networks using a preferential attachment model, i.e., the probability of a new node creating a connection with an existing node is proportional to the number of connections that the existing node has. This would result in new nodes tending/preferring to form connections with highly connected nodes.
8. *Facebook348*, *Facebook414*^29^ are two social networks extracted from Facebook. Here the nodes represent users (friends) and the edges represent the different web-based social interactions (of liking, sharing, or messaging).
9. *ca-sandi_auths*^30^ is a collaboration network of 86 scientists at Sandia National Labs.
10. *fb-pages-food*^30^ represents Facebook pages of several food companies (collected in 2017) and how they mutually interact among each other.
11. *soc-tribes*^30^ is a network acquired from the study conducted on the tribes of central Highlands of New Guinea^31^. It shows the cultural-linguistic groups of that area and what similarities and differences exist among them.
12. *bn-cat-mixed-species_brain_1*^30,32,33^ is the neural connection networks (connectome) of cortical areas from the brain of cats.
13. *bn-macaque-rhesus_brain_2*^30,32^ represents the connectome that existed in the brain of rhesus macaque monkeys.
14. *bio-celegans*^23,30,33^ represents the connectome network of the Caenorhabditis elegans^34,35^.
15. *soc-firm-hi-tech*^30^ represents a network of friendships among employees of a small hi-tech computer firm.
16. *Circuits*^36^ represents electrical circuits networks, retrieved from (http://www.weizmann.ac.il/mcb/UriAlon/download/collection-complex-networks.
17. Aves-weaver-social^30,37^ represents animal social networks that represent the usage of the same nest chambers by several sociable weavers.
18. Bio-SC-TS^30,38^ is a high-precision gene network representation of gene-to-phenotype associations which resulted from the modified Bayesian integration of several data-type-specific networks.
19. CAG_mat72^30,39^ represents a Computer Algebra Group (CAG) matrix set aimed to solve a combinatorial problem.
20. ENZYMES123, ENZYMES8^30^ represent real-world examples of biological networks comprising regulatory interactions.
21. Reptilia-tortoise-network-(bsv, cs, fi, lm, mc, pv, sg)^30,40^ provides seven animal social networks that represent the interaction of desert tortoises. All networks were projected from a bipartite network type into single-mode tortoise nodes.

**Table 1:** Network properties of 29 networks used in the analysis; |*V*|: number of nodes in the network, |*E*|: number of edges in the network, *r*: Assortativity coefficient value, GCC & ACC: Global and average clustering coefficients, *ASP*: Average shortest path, *d*: diameter, and $\mathbf {D}$: graph density.

Name	|*V*|	|*E*|	*GCC*	*ACC*	*ASP*	*r*	*d*	$\text {D}$
Bn-macaque-rhesus_brain_2	91	582	0.2678	0.8601	1.8681	− 0.7698	3	0.1421
Karate	34	78	0.2557	0.5706	2.4082	− 0.4756	5	0.1390
*E. coli*	1565	3742	0.0155	0.2116	3.5791	− 0.3411	9	0.0031
Ca-sandi_auths	86	124	0.2721	0.4149	4.8140	− 0.2558	11	0.0339
Soc-firm-hi-tech	33	123	0.3875	0.6705	1.7689	− 0.2557	2	0.2330
Circuits1	122	304	0.0709	0.5656	1.9744	− 0.2487	3	0.0412
Bio-celegans	758	2025	0	0	3.7294	− 0.2233	11	0.0071
USAir97	332	2126	0.3964	0.6252	2.7381	− 0.2079	6	0.0387
Word adjacencies	112	425	0.1569	0.1728	2.5356	− 0.1293	5	0.0684
Polbooks	105	441	0.3484	0.4875	3.0788	− 0.1279	7	0.0808
Barabasi_albert_graph	500	1491	0.0322	0.0543	3.2335	− 0.0882	6	0.0120
Soc-tribes	17	76	0.6131	0.6488	1.4485	− 0.0792	2	0.5588
Dolphins	62	159	0.3088	0.2590	3.3570	− 0.0436	8	0.0841
fb-pages-food	620	2102	0.2226	0.3309	5.0887	− 0.0282	17	0.0110
bn-cat-mixed-species_brain_1	65	730	0.5747	0.6614	1.6995	− 0.0254	3	0.3510
ENZYMES8	141	133	0	0	4.3333	0.0161	10	0.0135
Reptilia-tortoise-network-sg	24	26	0.4390	0.2639	3.6538	0.0162	9	0.0942
Reptilia-tortoise-network-mc	15	28	0.6724	0.7094	1.6727	0.0408	3	0.2667
CAG_mat72	72	750	0.6541	0.7511	2.1291	0.0470	6	0.2934
Reptilia-tortoise-network-pv	35	66	0.5045	0.4884	2.4589	0.0583	6	0.1109
ENZYMES123	135	127	0	0	5.7098	0.0912	12	0.0140
Reptilia-tortoise-network-lm	45	106	0.3644	0.4345	2.6439	0.1181	6	0.1071
Aves-weaver-social	445	1335	0.5881	0.6685	4.4699	0.2000	12	0.0135
Facebook348	448	6384	0	0	3.0253	0.2227	10	0.0638
Facebook414	300	3386	0	0	3.1918	0.3064	8	0.0755
Reptilia-tortoise-network-bsv	136	374	0.3649	0.3335	3.7357	0.3254	10	0.0407
Reptilia-tortoise-network-cs	73	132	0.4158	0.3146	2.4022	0.3934	6	0.0502
Reptilia-tortoise-network-fi	787	1197	0.4199	0.2680	7.9334	0.4766	21	0.0039
Bio-SC-TS	636	3959	0.9137	0.4712	1	0.9211	1	0.0196

The networks are sorted according to the assortativity level; ($0 > r \ge -1$) for disassortative networks, ($1 \ge r > 0$) for assortative networks.

### Formal problem setting

Let $G=(V, E)$ be an undirected graph, with a set of nodes (or vertices *V*) and a set of edges *E*, where each edge represents a relationship between two nodes. We excluded circles (or loops), repeated edges, and isolated nodes from the network. Assume *U* to be the set of all possible edges between all nodes within the network. Let *L* represent the set of missing links of the graph *G*, i.e. $L = U - E$. The link prediction aims at predicting possible non-existing links between nodes at a future time slot ($t_{i+1}$) given the graph structure at the current time slot ($t_i$). The link prediction model uses the different topological and structural features of the graph at the current time slot that may contribute to the forecasting of future links. Therefore, the different link prediction models compute the possibility of having edges depending on a pre-defined scheme. Several criteria exist for the link prediction models as discussed next.

Each network dataset $G=(V, E)$ is divided into two subgraphs with non-overlapping edge sets: $G^T$ and $G^P$, or the training and probe graphs, respectively. $G^T$ is obtained by randomly sampling edges (and their nodes) from the original network *G*; let’s refer to edges in $G^T$ as $E^T$. $G^P = G - G^T$, such that the probe graph comprises the remaining edges referred to as $E^P$. For experimental purposes, 80% of the edges in *G* go to $E^T$ and form $G^T$ and the rest go to $E^P$ to form $G^P$. Apparently, $E = E^T + E^P$, and nodes in both the training and probe graphs may overlap. Training graph $G^T$ will be the input to the link prediction model. The model will only process the local connectivity of the $G^T$ networks and predict the possibility of whether there will be an edge among the node pairs in the probe graph $G^P$ and form a new graph $G'$^41^.

### Proposed models

We assume that two nodes could form an edge if they satisfy one or both of the following:

1. Nodes *x*, *y* share similar neighborhoods.
2. Nodes *x*, *y* have different influence/impact levels within the network.

The proposed model depends on two basic concepts of complex networks: common neighbors and the degree of the nodes. Common neighbors refer to the number of nodes that exist as a neighbor between both *x* and *y*, see Eq. (10). The degree of the node may refer to the amount of (connections, influence, contribution, or power) of the node within the network. Node degree refers to the number of (edges/connections/relationships) a node has with other nodes. The degree of node *x* can also be interpreted by the number of neighbors, presented as $|\Gamma _x|$. We considered the node’s power/degree as a factor due to the fact that low-degree nodes tend to create a connection with high-degree nodes in a phenomenon known as rich becomes richer; however, nodes that can make *more* connections tend to form a cluster of nodes with each other such that they share the same range of middle to high degree neighborhood. Low-degree nodes have neither the tendency to form connections nor carry enough information about neighbors’ similarities. As a result, to measure the possibility for two nodes *x*, *y* to create a future link, we considered the difference in power (or influence) and the common neighborhood of the two nodes.

#### Node influence

We propose two measurements to compute the contribution or influence of a node: (1) global node’s influence (GI) and (2) local node’s influence (LI).

1. Global influence of a node (GI): In this model, the node’s degree is compared to the average degree of the network to reflect the global influence of the node within the network. Equation (1) depicts node *u*’s influence relative to the average degree of the network, ($I^G_u$).
  1 $$\begin{aligned} I^G_u = \frac{|\Gamma _u|}{\frac{1}{|V|}\sum \limits _{v \in V} |\Gamma _v|} \qquad u \in V \end{aligned}$$
2. Local influence of a node (LI): In this model, the node’s degree is compared to the average degree of the node’s neighbors to reflect the local influence of the node within its neighborhood area. Equation (2) depicts a node *u*’s influence with respect to the average degree of its neighbors, ($I^L_u$).
  2 $$\begin{aligned} I^L_u = \frac{|\Gamma _u|}{\frac{1}{|\Gamma _u|}\sum \limits _{v \in \Gamma _u} |\Gamma _v|} \qquad u \in V \end{aligned}$$

Based on node influence, we present three groups of models (discussed hereafter) to measure the possibility of having a link between two nodes: (1) extended preferential attachment models, (2) dissimilarity models and (3) similarity-based models.

#### Extended preferential attachment model

A well-known phenomenon of rich becomes richer [i.e., preferential attachment, see Eq. (13)] is where low-degree nodes tend to form a connection with highly connected nodes, especially in networks that follow a power law degree distribution. However, nodes may also tend to create a connection with other nodes based on the degree of influence within the network. Future links can be calculated using the same methodology as the preferential attachment, but using the node’s influence instead of the node’s degree. Herein, the node’s influence towards attachment can utilize the node’s global influence ($I^G_u$) or its local influence ($I^L_u$), $u \in V$. Preferential attachment using global influence (PAGI) and preferential attachment using local influence (PALI) is used for scoring the potential for having a link/edge between $x, y \in V$ using Eqs. (3) and (4).

3 $$\begin{aligned} S^{PAGI}_{x, y}= & {} I^G_x * I^G_y \end{aligned}$$

4 $$\begin{aligned} S^{PALI}_{x, y}= & {} I^L_x * I^L_y \end{aligned}$$

#### Dissimilarity models

The second model can be viewed as an extension to the preferential attachment where low-degree nodes tend to establish a connection/link with higher-degree nodes. Given this scenario, the potential for having an edge is increased as the difference in power/influence between the two nodes is increased. Herein, the absolute difference of either global influence (*GI*) or local influence (*LI*) has been used as a way to measure the possibility of establishing a link between the two given nodes. Therefore, as the difference (or dissimilarity) in power increases, there will be a higher chance of creating a link. Dissimilarity-based attachment using global influence (DAGI), and dissimilarity-based attachment using local influence (DALI) are used for scoring the potential of having a link between $x, y \in V$ using Eqs. (5) and (6).

5 $$\begin{aligned} S^{DAGI}_{x, y}= & {} |I^G_x - I^G_y| \end{aligned}$$

6 $$\begin{aligned} S^{DALI}_{x, y}= & {} |I^L_x - I^L_y| \end{aligned}$$

#### Similarity models

Herein, distance is mostly used to check the degree of similarity of two given items; the high distance value means less similarity and vice versa. Thus, the third model can be viewed as an inverse of the dissimilarity-based attachment using global influence (inDAGI), and dissimilarity-based attachment using local influence (inDALI), see Eqs. (7) and (8).

7 $$\begin{aligned} S^{inDAGI}_{x, y}= & {} \frac{1}{S^{DAGI}_{x, y}} = \frac{1}{|I^G_x - I^G_y|} \end{aligned}$$

8 $$\begin{aligned} S^{inDALI}_{x, y} = \frac{1}{S^{DALI}_{x, y}}= & {} \frac{1}{|I^L_x - I^L_y|} \end{aligned}$$

These three different models consider the local and global influence of the nodes (an extension of the node’s degree) in the prediction of a connection between the two nodes. These metrics can also contribute to the local similarity-based metrics (such as common neighbors) in forming the connection. The resultant combined link prediction model that calculates the potential link score is given as follows:

9 $$\begin{aligned} S_{x, y} = \alpha . S^{CN}_{x, y} + (1- \alpha ) . S^{Model}_{x, y} \qquad Model \in \{ PAGI, PALI, DAGI, DALI,inDAGI, inDALI\} \end{aligned}$$

A parameterized contribution of both of common neighbors and one of the proposed models would provide the final score. The $\alpha$ parameter ranges in [0.2, 0.4, 0.6, 0.8]. We used the $\alpha$ value to show the degree of the contribution that each measure has towards better overall link prediction. As the contribution of one measure increases, the contribution of the other will decrease.

### Baseline algorithms

We compared our proposed models (refer to Section “Proposed models”) with the following local similarity-based algorithms. Local similarity-based approaches use node neighborhoods to measure the similarity of each node with other nodes in the network. Local approaches are faster than non-local approaches and it is highly parallelizable and efficient for dynamic networks. However, all of the following algorithms have a computation complexity of $O(vk^3)$ except for the preferential attachment which has a computation complexity of $O(vk^2)$; *v* refers to the number of vertices (nodes) and *k* refers to the degree of the node. Most of these methods are well explained in Martinez et. al^13^.

1. *Common Neighbors*^42^ (CN) is the simplest and fundamental local technique. It measures the number of shared neighbors between two nodes *x*, *y*. A confirmed hypothesis^43^ shows that for two distinct nodes, there is a correlation between the number of shared neighbors and the probability of being connected. The formula for $S^{CN}_{x,y}$ is as follows:
  10 $$\begin{aligned} S^{CN}_{x,y} = |\Gamma _x \cap \Gamma _y| \end{aligned}$$
2. *Adamic-Adar Index*^44^ (AA) is another variation of common neighbors which measures the similarity between *x*, *y* by logarithmically penalizing the shared neighbors.
  11 $$\begin{aligned} S^{AA}_{x,y} = \sum \limits _{z \in \Gamma _x \cap \Gamma _y} \frac{1}{\log |\Gamma _z|} \end{aligned}$$
3. *Resource Allocation Index*^45^ (RA) is another variation of both common neighbors and the Adamic-Adar index which models the unit of resources between two unconnected nodes through neighborhood nodes. The number of resource units transmitted from node *x* using *x*’s neighbors and received by node *y* reflects the degree of similarity between *x*, *y*.
  12 $$\begin{aligned} S^{RA}_{x,y} = \sum \limits _{z \in \Gamma _x \cap \Gamma _y} \frac{1}{|\Gamma _z|} \end{aligned}$$
4. *Preferential attachment*^19^ (PA) is based on a premise that in a large set of real networks, node degrees tend to follow a power law distribution resulting in scale-free networks. The probability of having an edge between two nodes increases as their degrees increase.
  13 $$\begin{aligned} S^{PA}_{x,y} = |\Gamma _x| |\Gamma _y| \end{aligned}$$
5. *The Jaccard Index*^46^ (JA) is a widely used similarity measurement that measures the ratio of shared neighbors in the complete set of neighbors for two nodes.
  14 $$\begin{aligned} S^{JA}_{x,y} = \frac{|\Gamma _x \cap \Gamma _y|}{|\Gamma _x \cup \Gamma _y|} \end{aligned}$$
6. *Salton Index*^47^ (SA) is another related measure to the Jaccard index, which is mostly known as the cosine similarity. In several experiments, the Salton index has been shown to be approximately twice the Jaccard index.
  15 $$\begin{aligned} S^{SA}_{x,y} = \frac{|\Gamma _x \cap \Gamma _y|}{\sqrt{|\Gamma _x||\Gamma _y|}} \end{aligned}$$
7. *Sorensen Index*^48^ (SI) is a very similar method to the Jaccard index, used to compare the similarity between different ecological community data samples.
  16 $$\begin{aligned} S^{SI}_{x,y} = \frac{|\Gamma _x \cap \Gamma _y|}{|\Gamma _x| + |\Gamma _y|} \end{aligned}$$
8. *Hub Promoted Index*^49^ (HPI) measures the similarity between *x*, *y* by comparing the ratio of common neighbors of nodes *x*, *y* to the minimum degree of either node.
  17 $$\begin{aligned} S^{HPI}_{x,y} = \frac{|\Gamma _x \cap \Gamma _y|}{min(|\Gamma _x|, |\Gamma _y|)} \end{aligned}$$
9. *Hub Depressed Index*^49^ (HDI) measures the similarity between *x*, *y* by comparing the ratio of common neighbors of nodes *x*, *y* to the maximum degree of either node.
  18 $$\begin{aligned} S^{HPI}_{x,y} = \frac{|\Gamma _x \cap \Gamma _y|}{max(|\Gamma _x|, |\Gamma _y|)} \end{aligned}$$
10. *Local Leicht-Homle-Newman Index*^50^ (LLHN) is a model where the similarity between *x*, *y* nodes is measured as the ratio of common neighbors of the *x*, *y* nodes to the multiplication of neighbors of the *x*, *y* nodes.
  19 $$\begin{aligned} S^{LLHN}_{x,y} = \frac{|\Gamma _x \cap \Gamma _y|}{|\Gamma _x| |\Gamma _y|} \end{aligned}$$
11. *Cannistraci-Alanis-Ravasi-based variation of the resource allocation*^33,51^ (CAR) is a model, where two nodes are likelier to have a connection if their common neighbors share very strong inner-links, forming so-called “local-community LC”.
  20 $$\begin{aligned} S^{CAR}_{x,y} = \sum \limits _{z \in \Gamma _x \cap \Gamma _y} \frac{\Gamma _x \cap \Gamma _y \cap \Gamma _z}{|\Gamma _z|} \end{aligned}$$
12. *CH2-L2 Index*^52,53^ is a link prediction model that assigns a reward for the internal connectivity existing among common neighbors and penalizes outside connectivity.
  21 $$\begin{aligned} S^{CH2-L2}_{x,y} = \sum \limits _{i \in \Gamma _x \cap \Gamma _y} \frac{1 + C_i}{1 + O_i} \end{aligned}$$
  Here $C_i$ represents the number of neighbors of node *i* that exist in $\Gamma _x \cap \Gamma _y$, $O_i$ represents the number of neighbors of node *i* that do not exist in $\Gamma _x \cap \Gamma _y$ nor in *x* or *y*.
13. *CH2-L3 Index*^52,53^: very similar to CH2-L2 metric, this metric considers all three path lengths (two intermediate nodes *i*, *j*) between the targeted edge (*x*, *y*).
  22 $$\begin{aligned} S^{CH2-L3}_{x,y} = \sum \limits _{i \in \Gamma _x, j \in \Gamma _y} \frac{A_{i,j} \sqrt{(1 + \bar{C_i})(1 + \bar{C_j})}}{\sqrt{(1 + \bar{O_i})(1 + \bar{O_j})}} \end{aligned}$$
  Here $\bar{C_i}$ represents the number of links between node *i* and all the nodes that exist in the set of intermediate nodes on all 3-hop paths connecting nodes *x* and *y*, $\bar{O_i}$ represent the number of links between node *i* and all nodes that are not *x*, *y* nor the intermediate nodes on any 3-hop paths connecting *x* and *y*.

**Figure 1:**
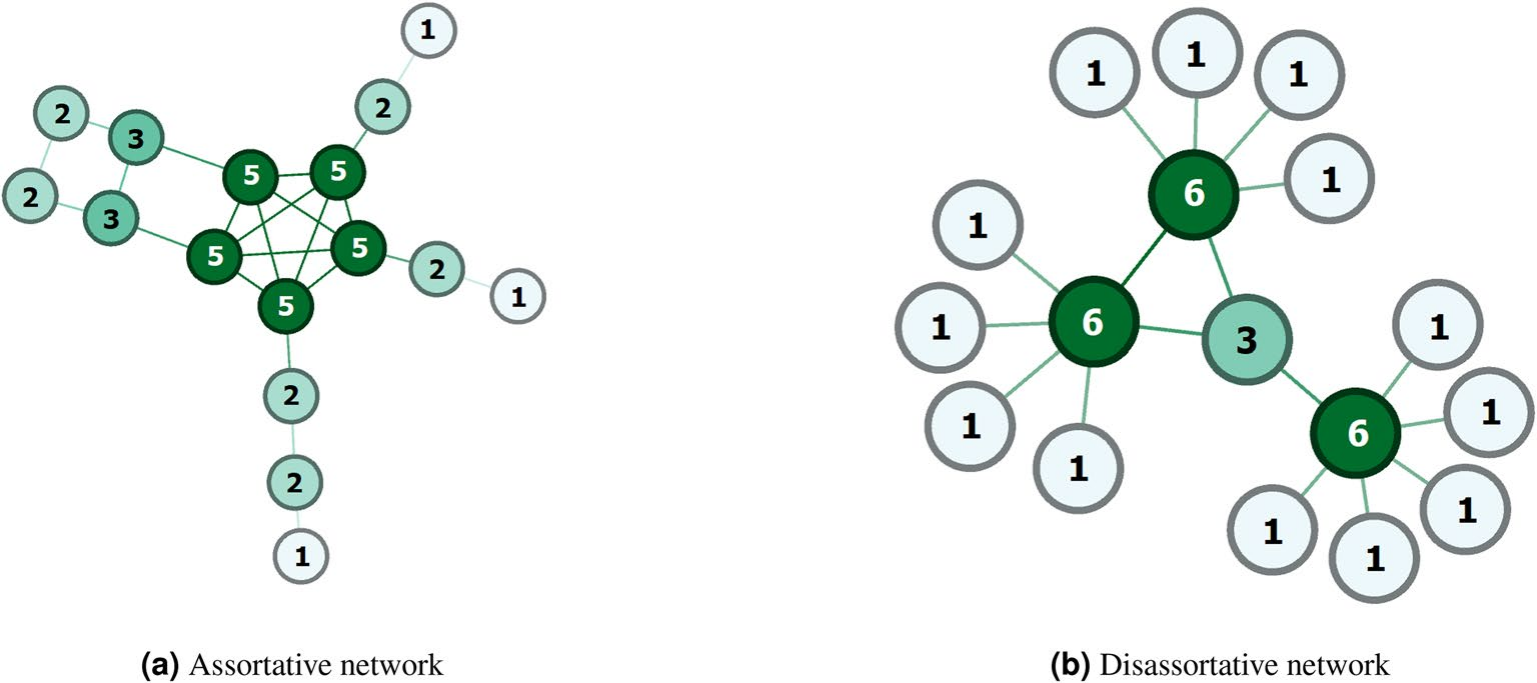
Level of assortativity for two networks. High-degree nodes are colored dark green while low-degree nodes are colored with a lighter color. Each node is labeled with its degree. (**a**) Assortative networks ($r = 0.6$) where high-degree nodes are attached to high-degree nodes and low-degree nodes are attached to low-degree nodes. (**b**) Disassortative networks ($r=-0.84$) where high-degree nodes are attached to low-degree nodes.

### Network assortativity

Assortativity, or assortative mixing, is a preference for the nodes to attach to other nodes that are similar in some way. Degree-based assortativity coefficient *r* of a network is measured as the Pearson correlation coefficient^54,55^ of the *degree* between all pairs of linked nodes, ranging from (− 1 to 1). Positive assortativity indicates a tendency of nodes to connect with other nodes of a similar degree. On the other hand, a negative correlation suggests that connections are more likely to exist between node pairs of dissimilar degrees.

23 $$\begin{aligned} r = \frac{\sum _{ij}(A_{ij}-k_i k_j/2m)k_i k_j}{\sum _{ij}(k_i \delta _{ij}-k_i k_j/2m)k_i k_j} \end{aligned}$$

Here, *A* is the adjacency matrix of the network, $k_i, k_j$ is the degree of node *i*, *j* respectively, $\delta _{ij}$ is Kronecker delta. Equation (23) is an example of a Pearson correlation coefficient where it has covariance in the numerator and a variance in the denominator. Figure 1 shows two networks of assortative and disassortative types.
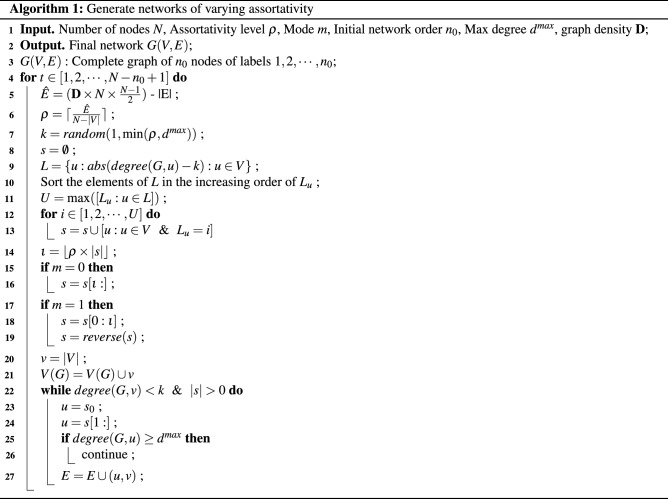


#### Generation of networks of varying assortativity

As discussed in Section “Introduction”, we extend the Monte Carlo sampling approach presented by Zhou et al.^21^ to generate assortative and disassortative networks of a given order. While Zhou et al. constrain the degree distribution, we constrain the graph density of the final network. Since the degree distribution affects the assortativity of a network, constraining it may restrict the approach from achieving the necessary level of assortativity (Algorithm 1). The algorithm takes as input the following: the number of nodes in the final generated network *N*, assortativity level $\rho$, mode *m* (equal to 0, 1 for assortative and disassortative, respectively), initial network order $n_0$ and maximum node degree in the final network $d^{max}$ with graph density $\mathbf {D}$, and outputs the undirected network *G*.

**Figure 2:**
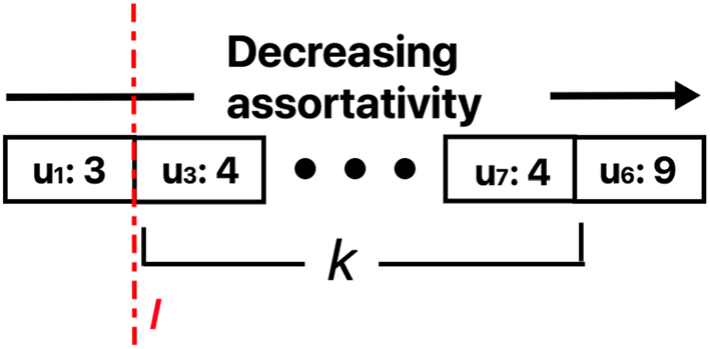
For each newly added node *v*, hash table *L*, where $L_u$ is the absolute difference between the degree of new node *v* and node *u*. The keys in *L* are arranged in the increasing order of $L_u$. The parameter $\iota$ marks the offset that controls the level of assortativity.

As shown in Algorithm 1, the final complete network *G* is initialized with $n_0$ nodes. Subsequently, new nodes (*v*) are added to the network iteratively as follows. In lines 5–7, we calculate $\hat{E}$ as the difference between the number of edges in *G* and the number of edges needed to achieve graph density $\mathbf {D}$. If $\hat{E} = 0$ the edges count requirement has already been met; otherwise surplus links are necessary. Specifically, once the surplus edge condition $\hat{E} = 0$ is met, the assortative network generation algorithm goes to the next new node. We calculate $\rho$ as the ratio of the surplus edges to be added and the number of nodes left to be added, i.e., $\rho = \lceil \frac{\hat{E}}{N - |V|}\rceil$, and assign the degree of new node *v* as $k = random(1, \min ({\rho , d^{max}}))$. Next, we generate a hash table *L* that indexes each node $u \in V$ by the absolute difference between its degree and *k*, i.e., $abs(degree(G, u) - k)$ (lines 8–13). As depicted in Fig. 2, *u*-th element in the hash table *L* is the absolute difference between the degree of new node *v* and node *u*, such that, the lower the difference, the more similar are nodes *u* and *v*. For every new node *v*, the idea is to choose a neighbor *u* with a (1) low $L_u$ or (2) high $L_u$ in order to generate a (1) assortative network or (2) disassortative network, respectively.

We input mode $m = 0$ to generate an assortative network, where higher $\iota \rightarrow |L|$ will make the network increasingly less assortative. Conversely, for disassortative network, *m* is set to 1, for which $\iota \rightarrow 0$ will make the network increasingly less disassortative (lines 14–19). Specifically, the algorithm controls the extent of assortativity, by sorting *L* in the increasing order of $L_u$ and introducing the assortativity level $\rho$. The parameter $\rho$ is necessary to determine $\iota$ which marks the index of nodes *u* in *L* that will be candidates for neighbors of *u*. Finally, in lines 22–27, we select *k* neighbors for node *v* ($u \in V$) depending on the choice of mode *m*, while enforcing the degree distribution, i.e., $\text {not} |[w: w \in V \& \, degree(G, w) = \, degree(G, u)]|) \le N \times P_{degree(G, u)}$. Finally, the network *G* is returned as output.

## Results

### Evaluation criterion

The link prediction models mentioned in the algorithms section measure a similarity score $S_{x,y}^m$ for every missing edge (*x*, *y*) in $G^P$, (*m* refers to the link prediction model in use). The resultant score estimates the possibility of having an edge between nodes *x*, *y* given its neighbors’ structures. If the similarity-score value $S_{x,y}^m$ equals or surpasses a threshold, then an edge between *x*, *y* is considered as predicted (or true positive *TP*) and otherwise rejected (false positive *FP*).

The range of similarity-score values that resulted from implementing the link prediction models varied even for the same graph. Therefore, to assess the performance of the different link prediction models on the given graph, the AUC (Area Under the receiver operating characteristic Curve) and AUPRC (Area Under Precision-Recall Curve) metrics are used. Given true positive (TP), true negative (TN), and false positive (FP) calculated on the true and predicted link labels, AUC and AUPRC are measured as follows^56–59^:

1. *Area under Curve* (AUC) is a metric that measures the extent to which the model is capable of distinguishing between classes. In the context of this paper, AUC measures the probability that a randomly chosen existing edge is given a higher similarity score $S_{x,y}$ than a randomly chosen non-existing edge. AUC is measured as follows:
  24 $$\begin{aligned} AUC = \frac{n_1 + 0.5 n_2}{n} \end{aligned}$$
  Here, $n_1$ is the number of times that the missing edge got a higher score than an unconnected edge, $n_2$ is the number of times when they are equal, and *n* is the number of observations done. If AUC = 0.5, then the score is generated from an independent and identical distribution. Thus, an AUC value closer to 1 indicates how much better the link prediction model is when compared to the prediction by chance. Overall, the higher the AUC, the better the model is at assigning the right labels to different classes.
2. *Area under Precision-Recall Curve* (AUPRC) captures the trade-off between *precision* and *recall*, where precision is equal to $\frac{TP}{TP + FP}$ and recall is $\frac{TP}{TP + TN}$. A high AUPRC suggests that the model exhibits both high precision and recall.

**Table 2:** Topological properties of a sample of the generated synthetic networks (of size 250) used in the analysis; Mean (and standard deviation of) GCC & ACC: Global and Average Clustering Coefficients, *ASP*: Average Shortest Path, *r*: Assortativity Coefficient value, *d*: diameter, and $\mathbf {D}$: graph density.

		*GCC*	*ACC*	*ASP*	*r*	*d*	$\mathbf {D}$
High density	High assortative	0.67 (0.04)	0.64 (0.02)	2.76 (0.23)	0.74 (0.03)	7.46 (1.44)	0.18 (0.01)
	Less assortative	0.35 (0.02)	0.29 (0.02)	2.03 (0.03)	0.3 (0.07)	4 (0.01)	0.19 (0.01)
	Less disassortative	0.18 (0.02)	0.2 (0.02)	1.86 (0.01)	− 0.28 (0.05)	3.19 (0.39)	0.19 (0)
	High disassortative	0.24 (0.02)	0.47 (0.04)	1.81 (0.01)	− 0.43 (0.02)	2.59 (0.49)	0.19 (0.01)
Low density	High assortative	0.54 (0.01)	0.17 (0.02)	11.92 (0.18)	0.88 (0.01)	24.97 (0.72)	0.01 (0)
	Less assortative	0.21 (0.03)	0.15 (0.03)	2.74 (0.24)	0.47 (0.05)	5.57 (0.64)	0.08 (0.02)
	Less disassortative	0.1 (0.01)	0.15 (0.02)	2.64 (0.05)	− 0.37 (0.01)	4.82 (0.39)	0.05 (0)
	High disassortative	0.11 (0.01)	0.15 (0.01)	2.66 (0.05)	− 0.51 (0.03)	4.73 (0.45)	0.05 (0)

**Figure 3:**
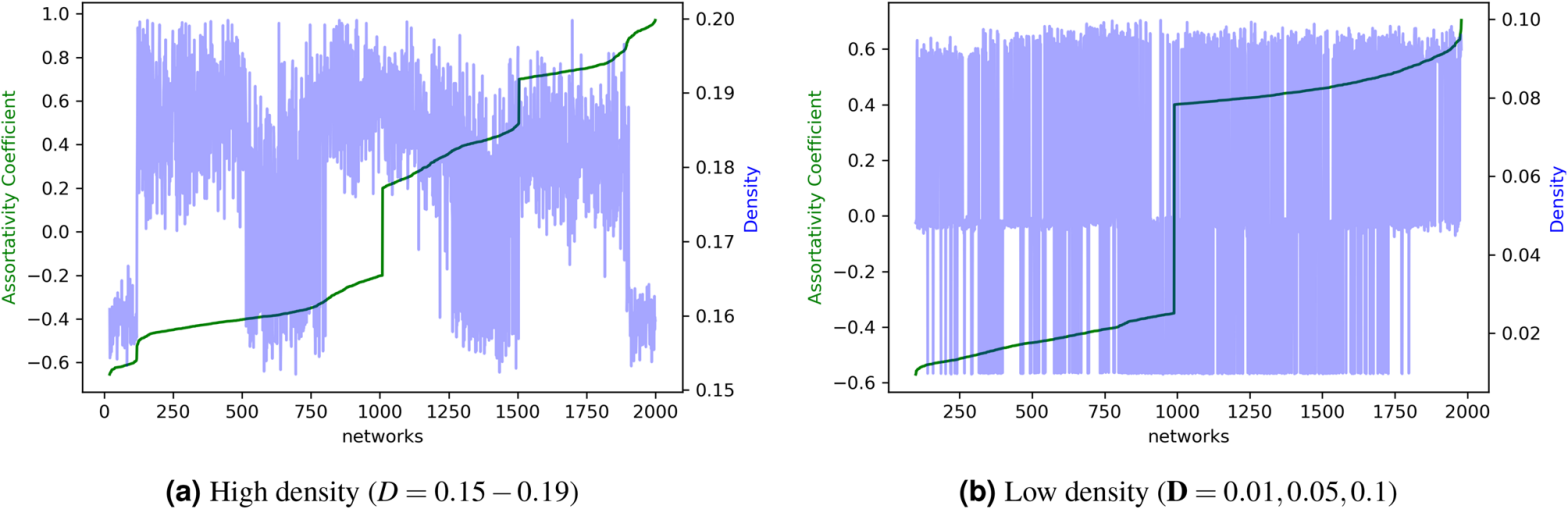
Assortativity coefficient value (*r*) and the corresponding density ($\mathbf {D}$) for all the synthetic networks ranked in the increasing order of assortativity. (**a**) shows the correlation between $r, \mathbf {D}$ using high density ($\mathbf {D}=0.15,0.19$). (**b**) shows the correlation between $r, \mathbf {D}$ using low density ($\mathbf {D}=0.01,0.05,0.1$). (In each figure, the first 1000 networks correspond to disassortative (or mode $m = 1$), while latter 1000 networks are for assortative (or $m = 0$)).

### Generation of assortative and disassortative networks

Algorithm 1 allows us to generate networks with varying assortativity (by modulating the assortativity level $\rho$). Table 2 shows the mean and standard deviation of the standard topological properties of 100 networks of size 250 nodes along with the level of assortativity and disassortativity and level of density. High assortative networks ($r > 0.7$) and less assortative networks ($0.25< r < 0.6$) are generated with $\rho = 0$ and $\rho = 0.2$, respectively. Conversely, high disassortative networks ($r < -0.45$) and low disassortative networks ($-0.40< r < -0.25$) are generated with $\rho = 1.0$ and $\rho = 0.8$, respectively. A correlation analysis is conducted between the graph density ($\mathbf {D}$) and network assortativity (*r*) for networks created by the generative algorithm. As shown in Fig. 3, no relationship exists between $r, \mathbf {D}$ on both high density networks (Fig. 3a) and low density networks (Fig. 3b).

**Figure 4:**
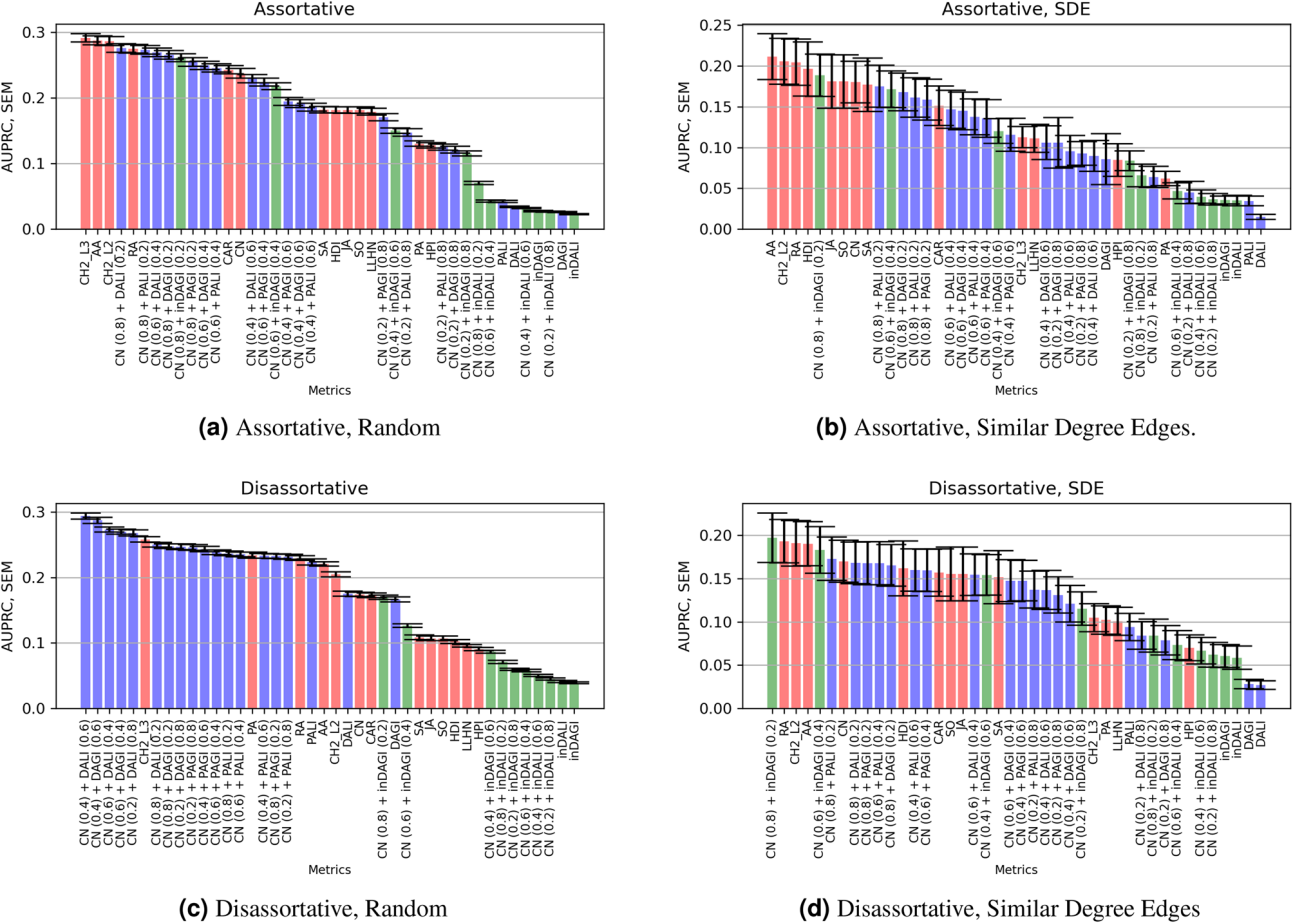
Performance of the LP models (i.e., the AUPRC values) implemented on the standard networks using random edges sampling (**a**, **c**), descending Similar Degree Edges (**b**, **d**). Assortative networks are shown in (**a**, **b**), and disassortative networks are shown in (**c**, **d**). Refer to Table 1 for details on the assortativity group each standard network belongs to. Red bars refer to standard LP models, blue bars refer to dissimilarity-based metrics along with their extensions and green bars refer to similarity-based metrics along with their extensions.

In addition to the standard sampling criteria that have been used in the literature for dividing the network edges into training ($E^T$) and testing ($E^P$), we apply another edge sampling criteria (termed *similar degree edges, SDE* criteria) to evaluate the performance of the different categories of the link prediction models. We collect the edges that have nodes with the same degree, sorted by the difference of degrees. This results in a descending list of edges, ranked in the decreasing order of score equal to the absolute value of the difference of nodes’ degree, i.e., $||\Gamma _x|-|\Gamma _y||$. We have shown in Section “The effect of edge sampling” that the removal of similar degree links results in disassortative training networks. Therefore, this sampling criteria represents a worst-case scenario where the metrics are used to predict edges among similar degree nodes despite being trained on disassortative networks.

### Cross validation on the synthetic networks

We implement the following cross-validation strategy. Unless otherwise stated, link prediction results reflect 50 runs (with 25 folds each) on 100 networks each of order 100, 200, 250, 500, and 1000 nodes.

1. For each of the 25 folds, the network set was randomly divided into 80% training and 20% testing set of networks. A network participates in either the training or the testing set.
2. The resultant evaluation values of the different link prediction models were collected and averaged resulting in an average over 25 folds $\times$ 50 runs.

### Prediction on standard networks

Standard networks are divided based on the assortativity coefficient *r* into assortative ($1 \ge r > 0$) and disassortative networks ($0 > r \ge -1$), see Table 1. Figure 4 shows the accuracy of the different link prediction metrics on assortative and disassortative networks, measured in terms of Area Under the Precision-Recall Curve (AUPRC). Figure 4a and b show the AUPRC for the assortative network group for random and similar degree edge removal, respectively, where local similarity-based metrics (AA, RA, and CH2-L2), relying on common neighborhoods, perform better than other metrics.

In the case of disassortative networks using random sampling of Fig. 4c, we also noticed that several weighted forms of the combined dissimilarity-based model of (CN+DALI, CN+DAGI) perform better than other local similarity models. Most of the standard and combined link prediction models that use local similarity (such as SA, JA, SO, CN+inDAGI, CN+inDALI) perform poorly. In Fig. 4d, we note that (CN+inDAGI) outperformed other metrics, using a similar degree of edge removal. We have not shown the results for preferential attachment using global influence (or PAGI as discussed in Section “Proposed models”). This is because it has a very similar formulation (i.e., the PAGI score is equivalent to the PA score divided by a constant) and yields the same accuracy as the preferential attachment (PA) metric for both standard and synthetic networks. However, we have reported high accuracy for the combination of PAGI with the common neighbor (CN).

### Prediction on synthetic networks

**Figure 5:**
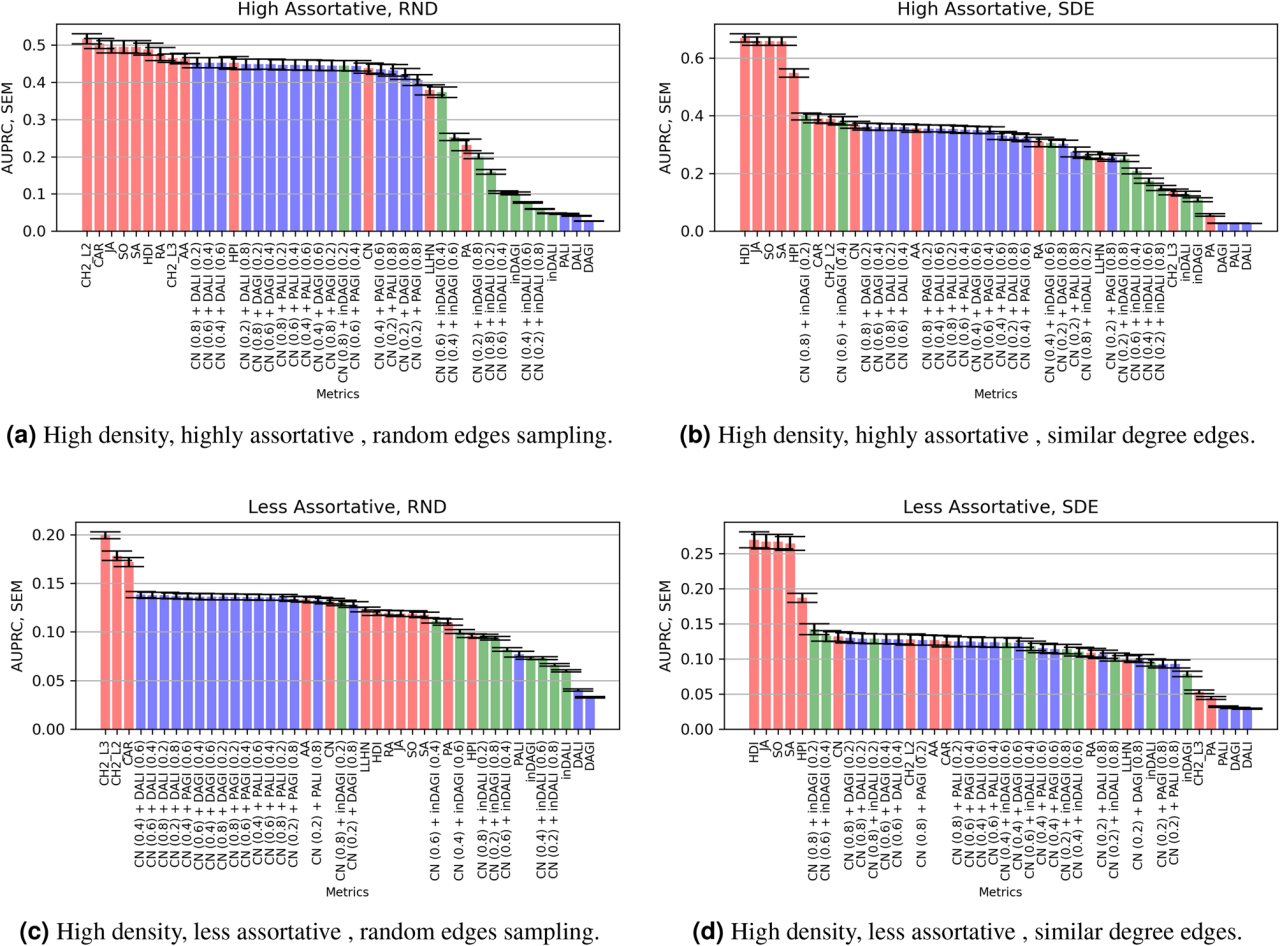
Performance of the LP models (i.e., the average AUPRC values) implemented on the high density, assortative synthetic network sets of size (100, 200, 250, 500, and 1000 nodes), 500 networks each. See Table 2 for high and less assortativity coefficient *r* ranges, respectively. Red bars refer to standard LP models, blue bars refer to dissimilarity-based metrics along with their extensions and green bars refer to similarity-based metrics along with their extensions.

**Figure 6:**
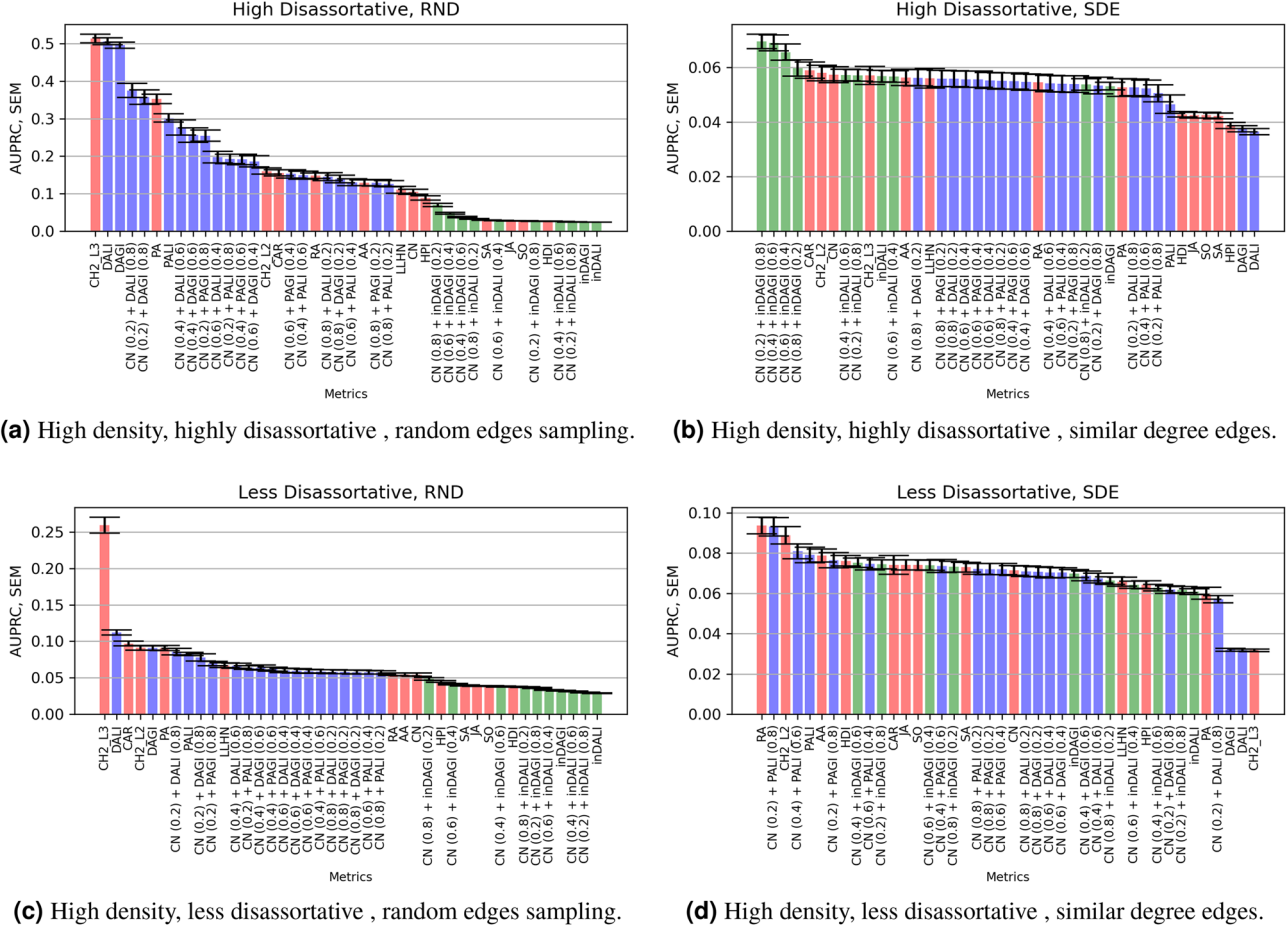
Performance of the LP models (i.e., the average AUPRC values) implemented on the high density, disassortative synthetic networks of size (100, 200, 250, 500, and 1000 nodes), 500 networks each. Table 2 depicts the high and less disassortativity coefficient *r* ranges, respectively. Red bars refer to standard LP models, blue bars refer to dissimilarity-based metrics along with their extensions and green bars refer to similarity-based metrics along with their extensions.

Figures 5 and 6 summarise the AUPRC results by implementing the link prediction metrics on the assortative and disassortative, high density ($\mathbf {D}=0.15,0.19$) synthetic networks, respectively. (The corresponding AUC results for the synthetic networks have been shown in Supplementary section 1 and section 2. Furthermore, the AUPRC results for the low-density networks (with density $D = \{0.01, 0.05, 0.1 \}$) have been shown in Supplementary Section 3).

#### Assortative networks analysis

Like in the standard assortative networks (discussed in Section “Prediction on standard networks”), the local similarity measures, namely CH2-L2, CH2-L3, Jaccard, HDI, and CAR, once again exhibit high accuracy for both random as well as similar degree edge sampling (see Fig. 5). The superior performance of local similarity-based metrics that rely on shared neighbors suggests that the similar degree nodes in the assortative networks are strongly interconnected.

#### Disassortative networks analysis

Figure 6 shows the AUPRC scores for the disassortative networks. CH2-L3, DALI, and DAGI outperform other LP metrics for the highly disassortative network, followed by combined dissimilarity-based models (with low CN contribution). Unlike the assortative networks, most similarity-based models (along with combined models) show very low AUPRC performance. Interestingly, in the case of similar degree edges sampling, similarity-based models of (inDALI and inDAGI) show higher AUPRC performance than preferential attachment extension and dissimilarity metrics).

### Key observations from the prediction models

*First*, we find that CH2-L2 and CH2-L3 perform consistently well for assortative and disassortative networks. This is because, unlike local similarity-based LP metrics, these metrics explore larger neighborhoods around the nodes of interest. *Second*, we report that the combined LP metrics of common neighbors (CN) and inDAGI exhibit an improvement over local similarity-based metrics in standard assortative networks (see Fig. 4b). This shows that a combined influence of local and global neighborhoods can often be a better strategy for standard assortative networks. Also, since similar degree nodes tend to group together in assortative networks, the local similarity-based metrics that rely on common neighbors (such as AA, RA, CN, etc.) can emerge as good choices for LP metrics. *Third*, for synthetic networks, since nodes in assortative networks tend to have connections with similar degree nodes, the AUPRC of the local similarity-based metrics decreases with network assortativity. We find low (positive) assortativity to be associated with the improved performance of the dissimilarity metrics (DALI, DAGI, etc.) as well as the preferential attachment combined metrics (PAGI, PALI) with CN. This is most evident in case of random sampling, as depicted in Figs. 5a,c and 6a,c. *Fourth*, we find CN + inDAGI to perform well in case of similar degree node removal and disassortative networks. A combination of low CN and high inDAGI has proven to be effective, showing the importance of the global influence of nodes (measured by their degree) in determining their network connections. Two nodes seem more likely to be connected if they have similar degrees rather than neighbor-based similarity. Overall, we intuit that the combined models can be particularly useful for predicting connections in real-world networks (namely, biological, social, and technological networks) which are often disassortative in nature^60^.

**Figure 7:**
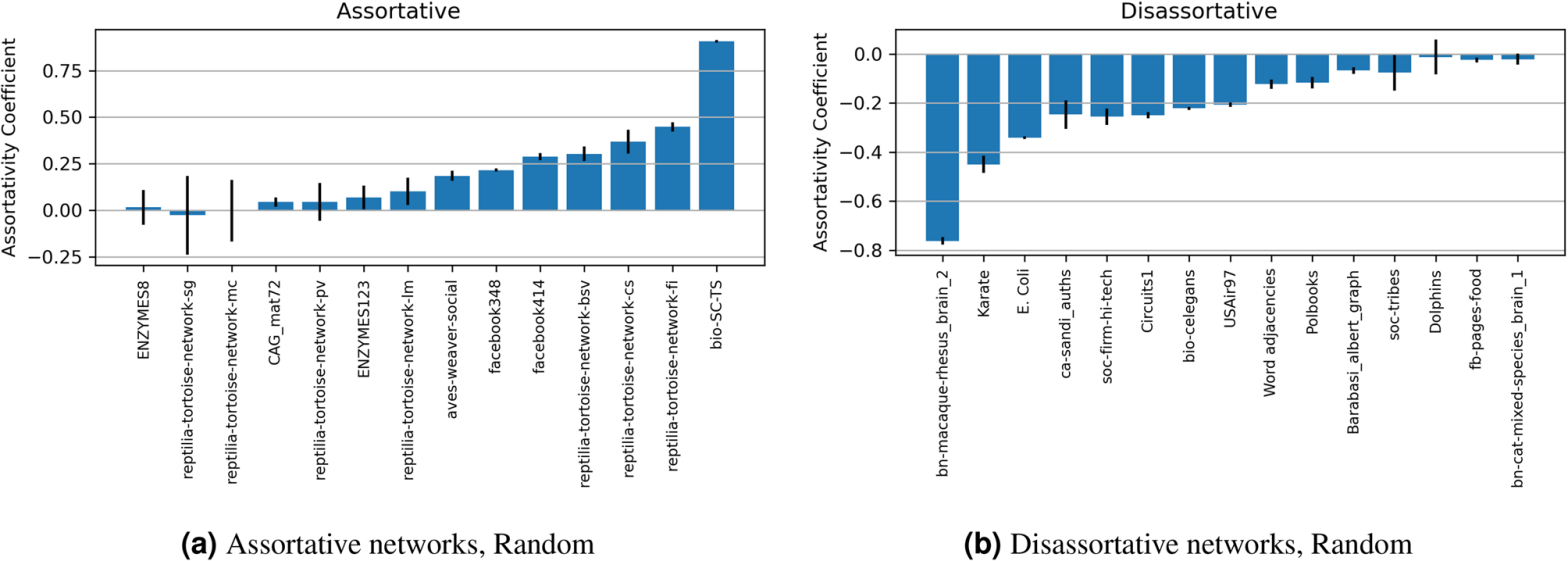
The average and standard deviation of the assortativity level of each of the standard networks after removing 20% of network edges at random for 30 separate runs. Edge removal of each run is implemented independently of other runs.

### The effect of edge sampling

The standard random sampling of edges guarantees that the type of edge to be selected is not biased and that no specific global or local feature of the network is targeted. Therefore, we observe that the assortativity coefficient *r* for the different standard networks maintained a close distance (small error ranges) with future sampling. Figure 7a reports the values of the assortativity coefficient *r* for each of the given networks such that we removed (20%) of the edges 30 times.

On the other hand, similar degree edges removal (or sampling) guarantees that the edges to be selected are determined by the similarity in degree of the two nodes at the specified edge. It is noteworthy that the two nodes are not necessarily similar in neighbors (as in common neighbors) or other standard local similarity-based metrics. Intuitively and after the removal of similar degree edges, the resultant network would have less assortativity coefficient *r* value, as can be seen in sFigure 7b, where we report *r* for standard networks after (20%) edges removal of the top similar degree ones, and for 30 times. We observe that the assortativity coefficient *r* is effected in a descending fashion, and gradually converts the network into disassortative networks. We can assume that the opposite is true such that the removal of dissimilar degree edges will increase the assortativity level.

Overall, the random and similar edge removal techniques are employed to demonstrate two aspects of the link prediction analysis. The random edge removal approach eliminates links without bias. Thus, the prediction occurs on probe networks very similar to the original networks (see Fig. 7a and refer to Section “Formal problem setting” for details on training and probe networks). On the other hand, as depicted in Fig. 7b, the similar edge removal scheme challenges the predictors by altering the assortativity coefficients of the training networks. In other words, the training is carried out on a disassortative network and the metrics are used to predict the possibility of links and degree-similar nodes.

**Figure 8:**
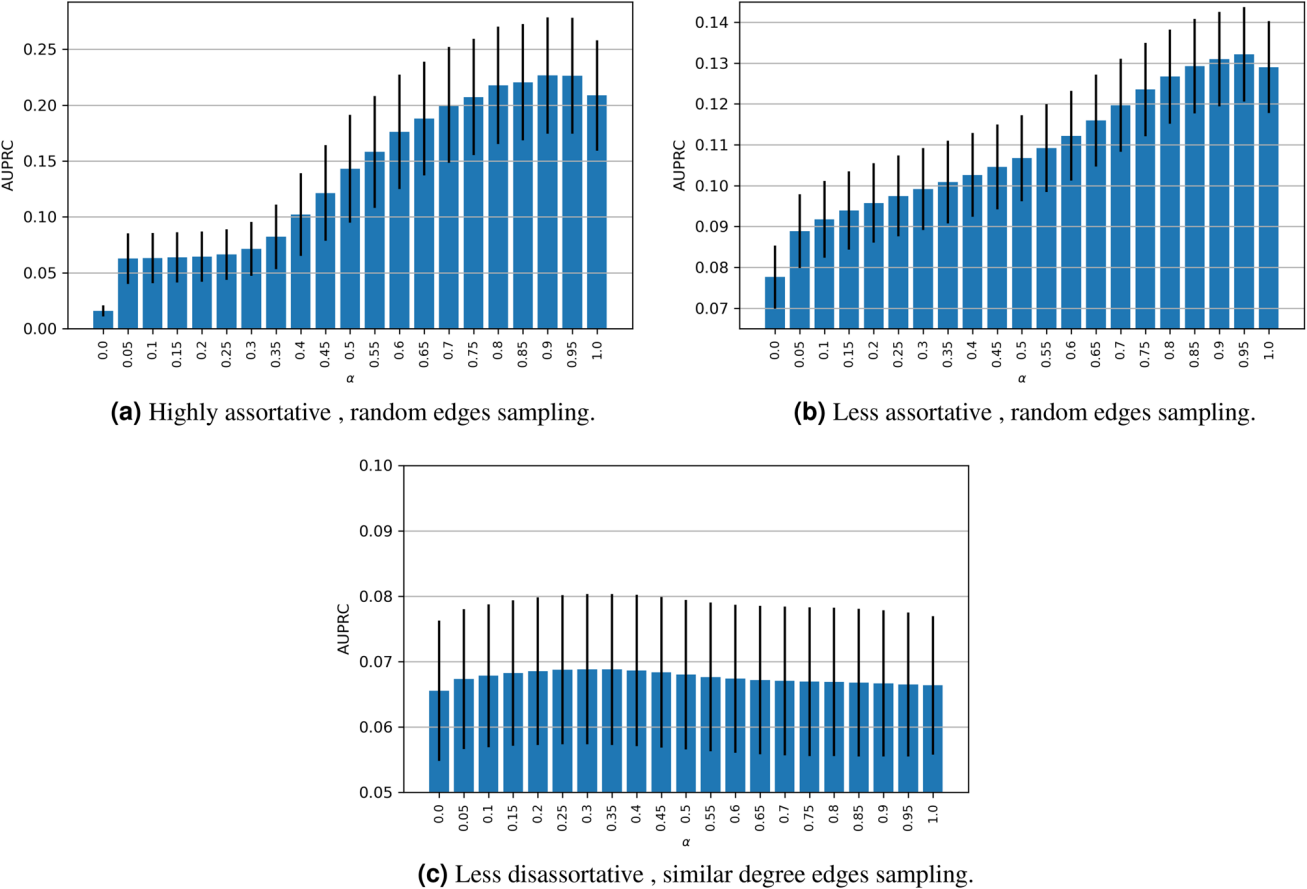
Exploratory analysis for $\alpha$. Showing the best AUPRC performance of the contribution of both of *CN* and *inDAGI* in ($S_{x, y}^{CN\_inDAGI}$), used on the 100 synthetic networks (of size 250), with different weights ($\alpha$).

### Exploratory analysis of $\alpha$ and the combined model

To better understand the impact of ($\alpha$) in the combined model, we reported the results that were collected from different simulations by using $\alpha = [0, 0.05, 0.1, \cdots , 1]$. We have already noticed the effect that *r* plays along with the two edge sampling approaches (Random, and SDE). Also, we have demonstrated that the combined model is constructed by two terms: common neighbors and either one of the proposed metrics of 1) similarity models (inDAGI, inDALI), 2) preferential attachment extension models (PAGI, PALI) and 3) dissimilarity models (DAGI, DALI). As the assortativity coefficient value *r* increases, the combined model tends to incorporate similarity metrics, i.e., CN + inDAGI and CN + inDALI, putting high weight on the CN model. Similarly, as the assortativity coefficient value *r* decreases (to disassortativity level), the combined model tends to incorporate dissimilarity metrics, i.e., (CN + DAGI and CN + DALI), putting high weight on the dissimilarity metrics.

However, in order to find the effect of ($\alpha$) on the combined model at high *r*, we particularly report additional analysis on the best similarity metrics of (CN + inDAGI) by considering a range of discrete values of $\alpha$. If $\alpha =0$, we consider 100% of the *inDAGI* value. Likewise, if $\alpha =1$, then we consider 100% of the *CN* value, see Fig. 8. We count the average AUPRC value (with its standard deviation) for the 500 networks of size 200 nodes, using high assortative, and less assortative networks with random edges sampling. Also, we count average AUPRC values (with their standard deviation) using similar degree edges. These three models show the best AUPRC performance for CN + inDAGI. We notice that there is a marginal contribution of the (*inDAGI*) of 10% in the high and less assortative networks (refer to Fig. 8).

## Discussions

In this paper, we have attempted to identify metrics for link prediction based on network assortativity. As part of this task, we introduced an approach that generates networks of varying assortativity levels and proposed three different models for link prediction that measure the different link properties. These models are local dissimilarity-based models (DAGI, DALI), extended preferential attachment models (PAGI, PALI) and finally similarity models (inDAGI, inDALI). These link prediction models are then combined with the most standard local similarity-based metric of common neighbors to form the weighted combined models. We have also introduced an algorithm to generate assortative and disassortative networks of varying levels. We carry out extensive simulation experiments to demonstrate the contribution of several standard local neighborhood-based metrics along with the common neighbors in the composition of the accurate link predictors for most cases. Our dissimilarity-based models outperform most of the other models in link prediction. Although there is less association between the assortativity coefficient of the network *r* and the link prediction models, we were able to show high prediction accuracy of specific models for assortative and disassortative networks.

This work opens up a few interesting research directions. First, we shall employ the proposed similarity and dissimilarity metrics to predict links in large-scale social and biological networks. In addition to the assortativity levels, this analysis will take into account other node and link labels as well as the directionality of links. Second, Fig. 8 shows the effect of varying the weighing parameter $\alpha$ on the overall accuracy of link predictions. Going forward, we intend to leverage these findings to infer general rules that will inform the selection of the link prediction metrics contingent on the assortativity and relevant feature information (of the nodes and links). Specifically, the rules will be mined using adaptive optimization algorithms that will learn the right $\alpha$ that maximizes accuracy for myriad assortativity levels as well as other topological properties of networks. Moreover, we shall delve deeper into the relationship between graph density and network assortativity in Algorithm 1. This will involve finding a range of graph densities for which it is feasible to generate networks of a prespecified assortativity coefficient. This effort will be particularly useful to the community of social and biological network researchers who need to analyze and make inferences from diverse families of partially available network datasets.

## Supplementary Information

Supplementary Information.

## Data Availability

The datasets used, generated, and/or analyzed during the current study along with the associated code are available in the GitHub repository (https://github.com/almusawiaf2/Identifying-Accurate-Link-Predictors-based-on-Assortativity-of-Complex-Networks/).
