# Supplementary material for: Identifying accurate link predictors based on assortativity of complex networks
Source: Sci Rep. 2022 Oct 27;12:18107. doi: 10.1038/s41598-022-22843-4 (PMC9613685; doi:10.1038/s41598-022-22843-4)
Supplement: Supplementary file 1 — Supplementary Information. [file 41598_2022_22843_MOESM1_ESM.pdf]

Supplementary Information

Ahmad F. Al Musawi, Satyaki Roy, and Preetam Ghosh

1 Prediction on Synthetic Networks

Figures 1 and 2 show the AUC results for link prediction metrics on the assortative and disassortative synthetic networks. Note that RND and SDE stand for random and simple degree edge removals respectively. In high r assortative networks (Figure1), we observe that most local similarity-based models achieve higher performance than the rest of the models, in both RND and SDE. That is true for less assortative SDE as well. In less assortative networks, we noticed that the CN model combined with similarity and dissimilarity-based models (of inDAGI, PALI, DALI, and PAGI) outperformed other models. On the other hand, in the disassortative network (Figure 2), we observe that (DAGI, DALI, PALI, and PA) have outperformed other LP metrics, followed by combined metrics (of low CN contribution). Local similarity-based metrics of (JA, SO, SA, HDI, HPI, RA, AA, and CN), have a low AUC performance.

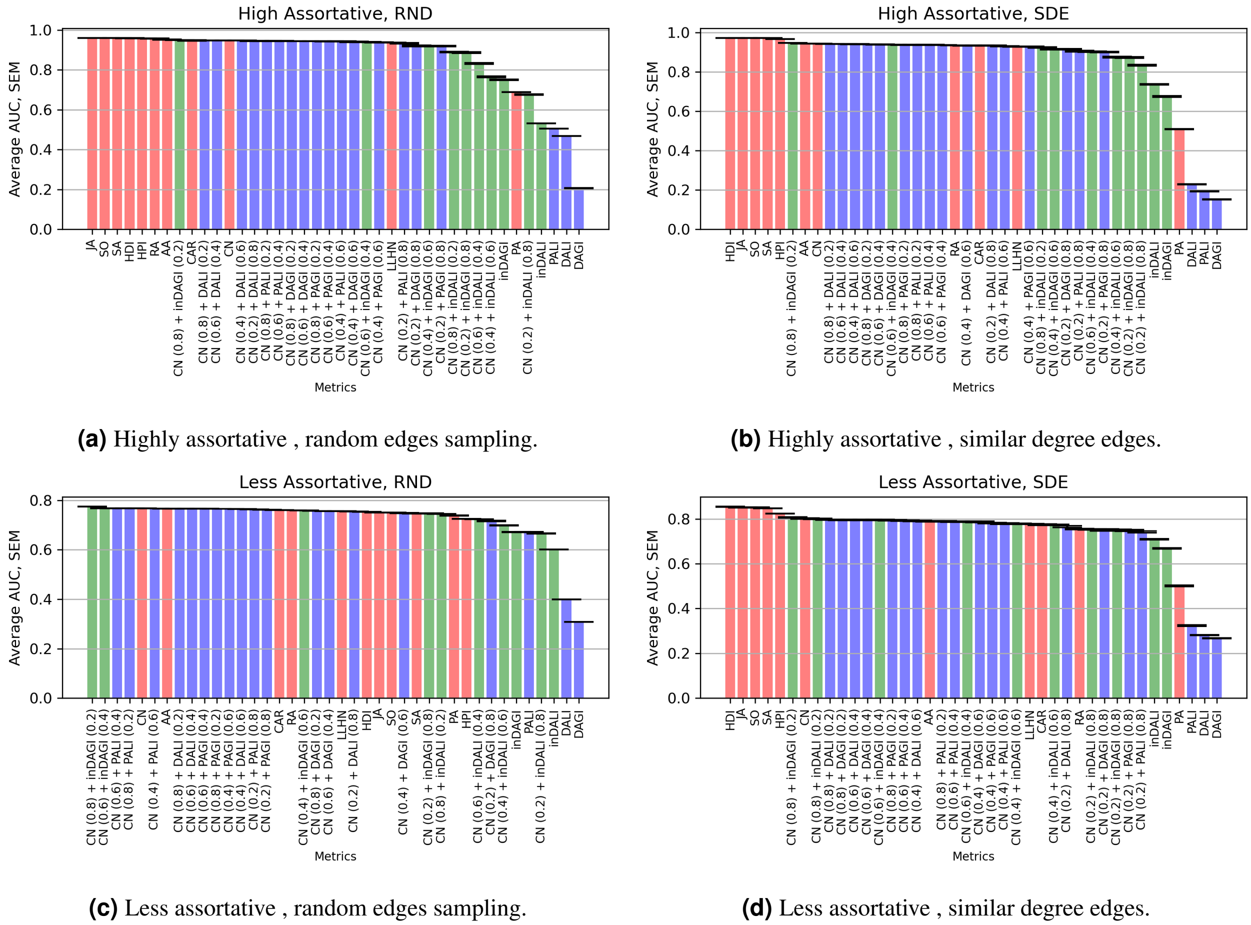

(a) Highly assortative , random edges sampling.
(b) Highly assortative , similar degree edges.
(c) Less assortative , random edges sampling.
(d) Less assortative , similar degree edges.

Figure 1. Performance of the LP models (i.e., the average AUC values) implemented on the synthetic assortative networks sets of size (100, 200, 250, 500, and 1000 nodes), 500 networks each.

2 Comparing against CH2 Scores

We carried out a separate analysis on highly assortative and disassortative synthetic networks, comparing the performance of the accuracy of the baseline link prediction metrics against CH2-L2 and CH2-L3 scores. Figs. 3a and 3b show that in addition to SO, SA and JA, CH2-L2 emerges as a promising metric for assortative networks; whereas CH2-L3 is the most effective for disassortative networks. This is because CH2-L2 rewards links among common neighbors, thereby predicting the existence of links among similar degree nodes in assortative networks with higher accuracy. On the contrary, for any potential edge (x,y)

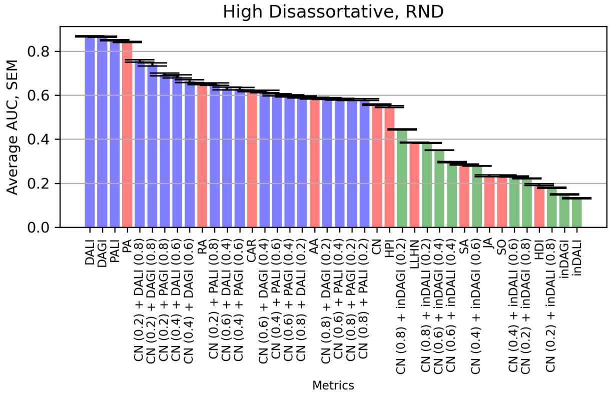

(a) Highly disassortative , random edges sampling.

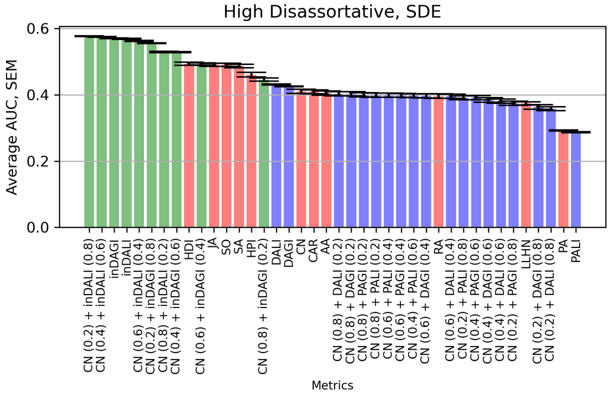

(b) Highly disassortative , similar degree edges.

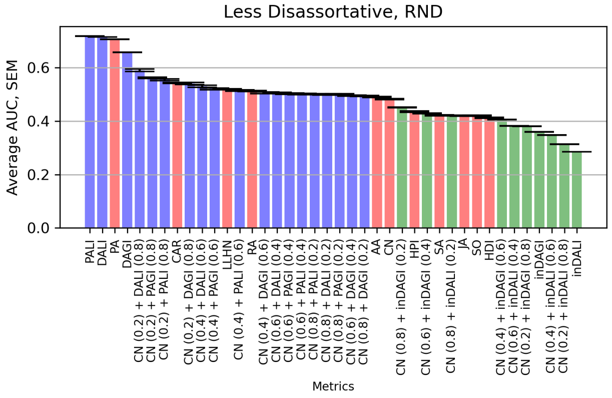

(c) Less disassortative , random edges sampling.

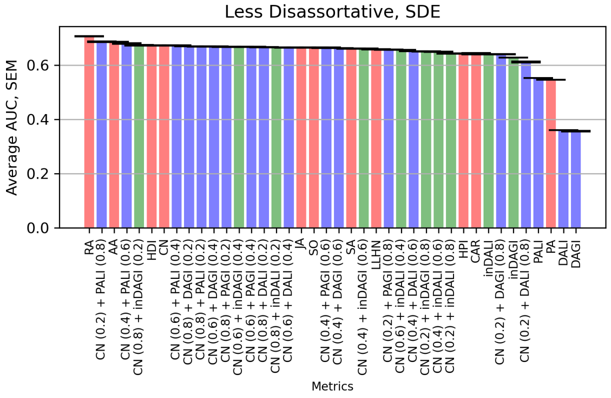

(d) Less disassortative , similar degree edges.

Figure 2. Performance of the LP models (i.e., the average AUC values) implemented on the synthetic disassortative networks of size (100, 200, 250, 500, and 1000 nodes).

CH2-L3 seeks links among neighbors of x or y and other nodes lying in the intermediate paths of 3 hops connecting x and y, better reflecting the topological principle of connectivity among dissimilar nodes in disassortative networks.

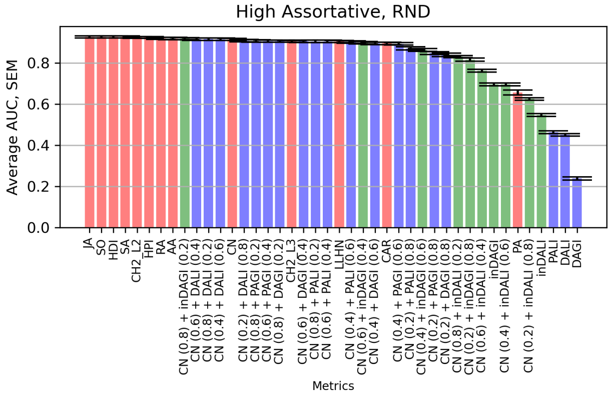

(a) Highly assortative , random edges sampling.

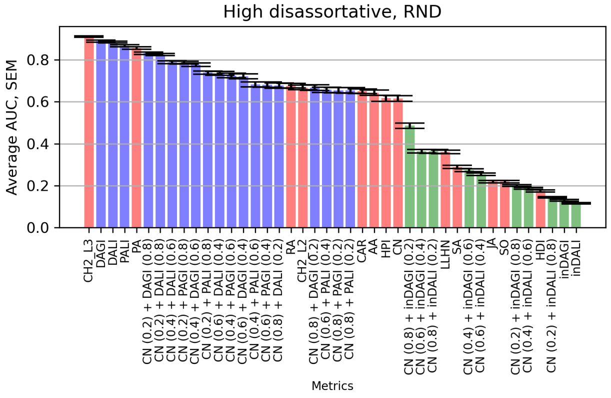

(b) Highly disassortative , random edges sampling.

Figure 3. Performance of the LP models (i.e., the average AUC values) implemented on the highly assortative and disassortative synthetic networks of size 100.

3 Performance on low-density networks

Figure 4 shows the performance of the different link prediction models on the generated synthetic networks of low density ($\mathbf{D} = \{0.01, 0.05, 0.1\}$). We do not see much difference in performance in comparison with those results of high density, in Figure 5(c, d) and Figure 6(c,d) of the main text.

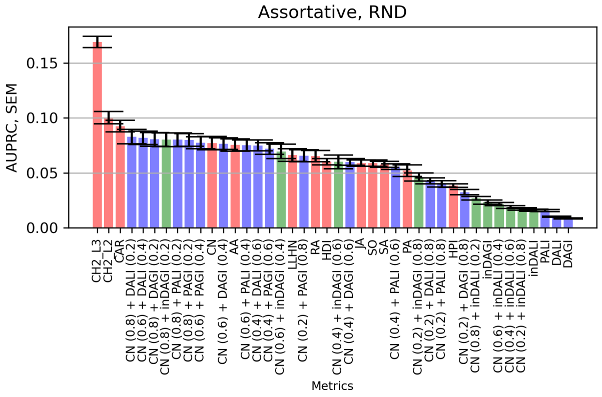

(a) Low density, assortative , random edges sampling.

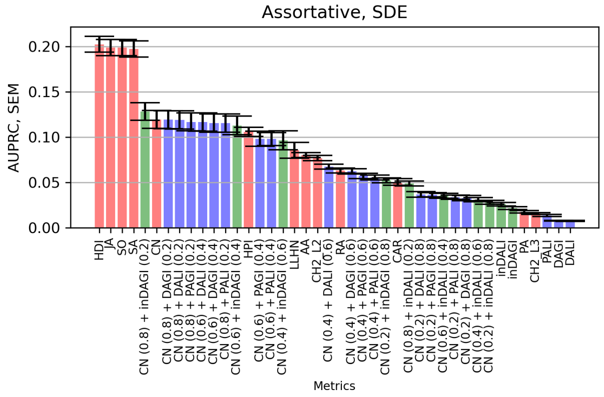

(b) Low density, assortative, similar degree edge removal.

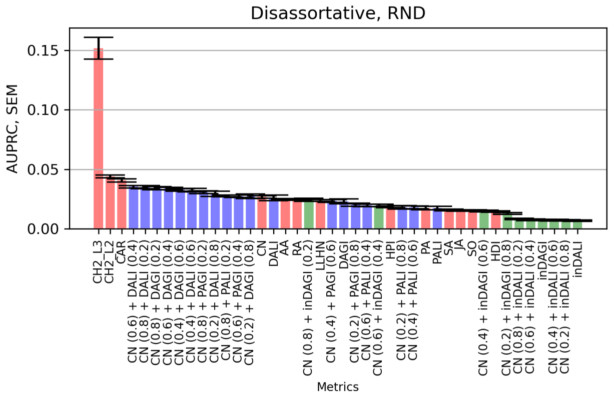

(c) Low density, disassortative , random edges sampling.

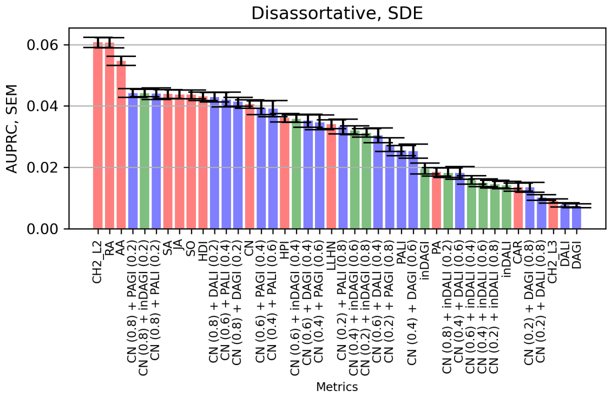

(d) Low density, disassortative , similar degree edge removal.

Figure 4. Performance of the LP models (i.e., the average AUPRC values) implemented on the low density, assortative and disassortative synthetic networks.
